# Training-Induced Plasticity of Auditory Localization in Adult Mammals

**DOI:** 10.1371/journal.pbio.0040071

**Published:** 2006-03-07

**Authors:** Oliver Kacelnik, Fernando R Nodal, Carl H Parsons, Andrew J King

**Affiliations:** **1**Department of Physiology, Anatomy, and Genetics, University of Oxford, United Kingdom; University of MarylandUnited States of America

## Abstract

Accurate auditory localization relies on neural computations based on spatial cues present in the sound waves at each ear. The values of these cues depend on the size, shape, and separation of the two ears and can therefore vary from one individual to another. As with other perceptual skills, the neural circuits involved in spatial hearing are shaped by experience during development and retain some capacity for plasticity in later life. However, the factors that enable and promote plasticity of auditory localization in the adult brain are unknown. Here we show that mature ferrets can rapidly relearn to localize sounds after having their spatial cues altered by reversibly occluding one ear, but only if they are trained to use these cues in a behaviorally relevant task, with greater and more rapid improvement occurring with more frequent training. We also found that auditory adaptation is possible in the absence of vision or error feedback. Finally, we show that this process involves a shift in sensitivity away from the abnormal auditory spatial cues to other cues that are less affected by the earplug. The mature auditory system is therefore capable of adapting to abnormal spatial information by reweighting different localization cues. These results suggest that training should facilitate acclimatization to hearing aids in the hearing impaired.

## Abstract 2

After training on a behaviorally relevant task, adult ferrets rapidly adapt to altered auditory localization cues by reweighing the less distorted spectral cues of the stimulus.

## Introduction

Accurate auditory localization relies on the detection and interpretation of spatial cues generated by the interaction between sound waves and the head and external ears [ 1]. Localization in the horizontal plane is based primarily on binaural disparity cues, interaural time differences (ITDs), and interaural level differences (ILDs), whereas spectral cues produced by the filtering properties of each external ear are used for making elevation judgments, discriminating between front and back, and, under certain conditions, for localizing sounds using one ear alone [ 2]. The precise cue values corresponding to each direction in space depend on the physical dimensions of the head and ears. Acoustical measurements in different species have demonstrated that these dimensions vary from one individual to another [ 3– 7], implying that listeners learn by experience to localize with their own ears. Indeed, psychophysical studies have shown that humans localize headphone signals that simulate real sound sources more accurately when these are derived from measurements made from their own ears than from the ears of other individuals [ 8, 9].

The plasticity of the auditory localization system can be probed by examining the capacity for adaptation to abnormal spatial cue values, produced either experimentally or as a consequence of pathological hearing. For instance, young barn owls [ 10, 11] and ferrets [ 12] can adapt to the altered binaural cue values produced by long-term blocking of one ear and learn to localize accurately despite the presence of an earplug. Moreover, this behavioral compensation is mirrored by adjustments to the midbrain auditory space maps found in these species [ 13, 14]. Adaptive plasticity appears to be restricted, however, in older animals [ 10– 13].

Attempts to investigate whether humans can adapt to abnormal binaural cues have produced conflicting results [ 15– 18]. On the other hand, it is clear that adult humans can accommodate altered spectral localization cues [ 19– 21]. For instance, vertical localization accuracy gradually recovers over the course of a few weeks after disrupting these cues by inserting molds into one [ 21] or both external ears [ 19]. It remains to be seen whether the mature auditory system possesses a similar capacity to learn a new correspondence between binaural cues and directions in space, what conditions are required in order to evoke this plasticity, or what the underlying mechanisms might be.

In this study, we examined the roles of behavioral training, vision, and error feedback in adaptation by adult ferrets to altered binaural cues produced by reversible occlusion of one ear. We show that rapid recovery of accurate spatial responses to sounds presented in the horizontal plane is possible when appropriate training is provided, that adaptation is not dependent upon feedback provided by the visual system, and that the basis for this plasticity involves learning to ignore the altered binaural cues and to attend selectively to those cues that are less affected by the earplug, namely low-frequency ITDs and the spectral cues provided by the unoccluded ear.

## Results

We investigated the effects of monaural occlusion on the ability of adult ferrets to localize broadband noise bursts in the horizontal plane by measuring the accuracy with which they approached the source of the sound. Figure 1 shows data obtained from one representative animal following presentation of either 1,000-ms ( Figure 1A– 1F) or 40-ms noise bursts ( Figure 1G– 1L) from each of 12 loudspeakers located at 30° intervals in the horizontal plane. These data are displayed both as stimulus-response plots, showing the distribution of responses made to each stimulus location ( Figure 1A– 1C and 1G–1I) and as polar diagrams in which the correct-score percentage is plotted as a function of speaker angle ( Figure 1D– 1F and 1J– 1L).

**Figure 1:**
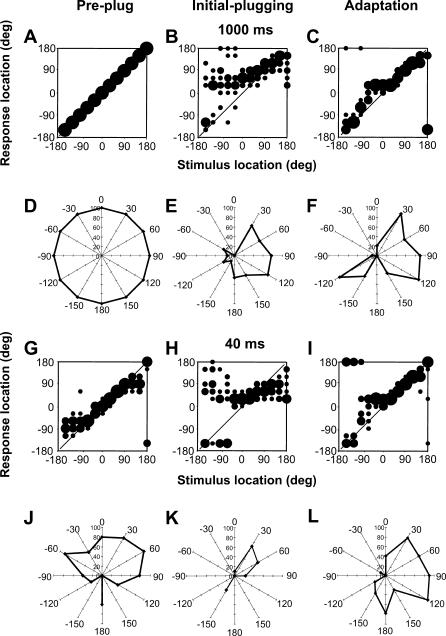
Effects of Monaural Occlusion on Ferret Auditory Localization (A–C and G–I) Stimulus-response plots for a representative animal, showing the distribution of responses (ordinate) as a function of stimulus location (abscissa). The size of the dots indicates, for each speaker angle, the proportion of responses made to different locations. Correct responses are those that fall on the diagonal line, whereas all other responses represent errors of different magnitude. (D–F and J–L) Polar plots showing the percentage of correct responses made to each of the 12 speaker locations. Each polar plot corresponds to the stimulus-response plot immediately above it. (A–F) Auditory localization responses to 1,000-ms noise bursts. (G–L) Responses to 40-ms noise bursts. The animal was tested before (A, D, G, J), on the same day that the left ear was plugged (B, E, H, K), and then every 6 d afterwards. Only the data obtained after 24 d of continuous plugging are shown (C, F, I, L).

Prior to occlusion of the left ear, this animal localized the longer duration stimuli perfectly, scoring 100% correct at all stimulus directions ( Figure 1A and 1D), but performed less well, particularly at lateral and posterior angles, at 40 ms ( Figure 1G and 1J). Plugging the left ear led to an immediate deterioration in performance at both stimulus durations ( Figure 1B, 1E, 1H, and 1K). The largest errors were made on the left, the side of the earplug, where very few correct responses were observed. The stimulus-response plots ( Figure 1B and 1H) show that the animal frequently mislocalized stimuli presented on the left to the opposite side of space. However, more errors were made at all speaker locations with the ear occluded, indicating the importance of binaural cues in this task.

In order to examine whether localization accuracy could be relearned using the altered cues, the earplug was left in place and the animal retested every 6 d. The data obtained at 24 d revealed a marked improvement in localization accuracy ( Figure 1C, 1F, 1I, and 1L). Performance for most stimulus locations on the right side, contralateral to the plugged ear, now approached that observed prior to the insertion of the earplug. Although relatively few correct responses were made to stimuli presented on the left ( Figure 1F and 1L), these were now mostly lateralized to the correct side of space ( Figure 1C and 1I), indicating that auditory localization accuracy had improved substantially since the left ear was first occluded.

### Role of Vision in Auditory Adaptation

It is well established that vision is used to coordinate spatial information provided by the different senses during development [ 14, 22– 24]. Moreover, it has been proposed that visual feedback about the accuracy of auditory localization could contribute to the adaptive changes observed after altering spectral cues in adult humans [ 19]. We therefore compared the effects of monaural occlusion in three animals that had been raised normally and three others that had been visually deprived by binocular eyelid suture from infancy. This allowed us to determine whether visual feedback is required for adaptation of auditory localization in adult ferrets. We calculated the unsigned errors at each speaker location and, because of the distribution of responses shown in Figure 1, combined these for the left and right sides of space. Data from individual animals are shown in Figure 2.

**Figure 2:**
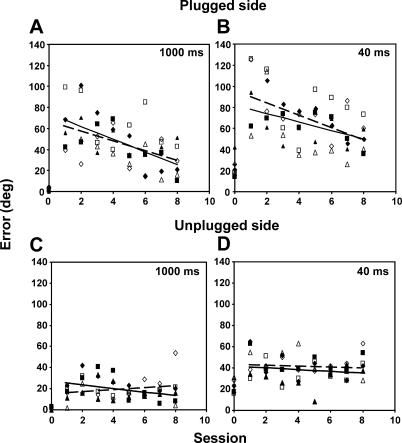
Adaptation Does Not Require Visual Feedback Data from three normally sighted (open symbols) and three visually deprived (filled symbols) ferrets showing the magnitude of the unsigned errors averaged across speaker location in the left (A and B; side of plugged ear) and right (C and D; side of open ear) hemifields. Each symbol represents a different animal. Data are shown for the session prior to insertion of the earplug (session 0) and for eight sessions, carried out at 6-d intervals, with the left ear occluded (sessions 1–8).

All six animals achieved a comparable level of performance prior to earplugging (session 0 in each panel of Figure 2), indicating that the loss of patterned visual cues did not impair their ability to localize sound. As expected from Figure 1, the magnitude of the errors increased, particularly on the ipsilateral side, following insertion of the earplug, but then declined steadily as the animals were retested every 6 d. The performance of these animals was significantly worse at 40 ms than at 1,000 ms ( *F*_1,4_ = 32.43, *p* < 0.01) and when the left ear was occluded ( *F*_1,4_ = 199.72, *p* < 0.01). To test the effect of visual experience, we computed linear regressions of mean unsigned error, averaged across subject and speaker location on each side of space (y), versus testing session with the earplug in place (x) for each group and each stimulus duration (see Figure 2 for regression lines). On the side of the earplug ( Figure 2A and 2B), all four regressions yielded significantly negative slopes (normal, 40 ms: y = 96.52–5.89x, R ^2^ = 0.50, *p* < 0.05; normal, 1,000 ms: y = 74.12–6.09x, R ^2^ = 0.73, *p* < 0.01; visually deprived, 40 ms: y = 82.01–3.98x, R ^2^ = 0.72, *p* < 0.01; visually deprived, 1,000 ms: y = 58.80–3.24x, R ^2^ = 0.73, *p* < 0.01). Using the method for comparisons of regression lines described by Snedecor and Cochran [ 25], we found no difference between the rate at which sighted and visually deprived animals adapted to the altered binaural cues (1,000 ms: *F*_2,12_ = 0.27, *p* = 0.77; 40 ms: *F*_2,12_ = 0.24, *p* = 0.79).

Our results show that adult ferrets can learn to reinterpret the abnormal localization cues produced by plugging one ear. Moreover, this process does not require a fully functioning visual system. When these ferrets were learning the task, they were rewarded for making a correct response. However, when the earplug was in place, the animals were rewarded for approaching any of the 12 sound sources. The observed improvement in localization performance shown in Figures 1 and 2 therefore indicates that error feedback is not necessary for adaptation. Indeed, no difference in the rate of adaptation was found when these animals were subsequently replugged and rewarded for making correct responses only (see following text).

### Learning Is Driven by Auditory Training

It is not clear from the data shown in Figure 2 or from previous studies of auditory spatial plasticity in humans [ 19] whether the degree and rate of adaptation are determined by the duration of exposure to altered spatial cues or by the extent to which those cues are actually used to localize sound. We addressed this issue by examining the effect of sound-localization training on the reacquisition of accurate spatial responses in a new group of six sighted ferrets. After the left ear was occluded, these animals were tested every day and their behavior reinforced by rewarding correct responses. Data from individual animals that were trained with either 1,000-ms noise bursts ( *n* = 3, Figure 3A and 3C) or 40-ms noise bursts ( *n* = 3, Figure 3B and 3D) showed that the localization errors initially caused by the earplug steadily declined at all stimulus locations. These improvements were observed both within and between sessions. Comparison of the overall error magnitude at the beginning and end of the 10-d period of monaural occlusion revealed a significant improvement in performance at both 1,000 ms (Wilcoxon matched-pairs signed-rank test: *p* < 0.02 in each case) and at 40 ms ( *p* < 0.05 in each case).

**Figure 3:**
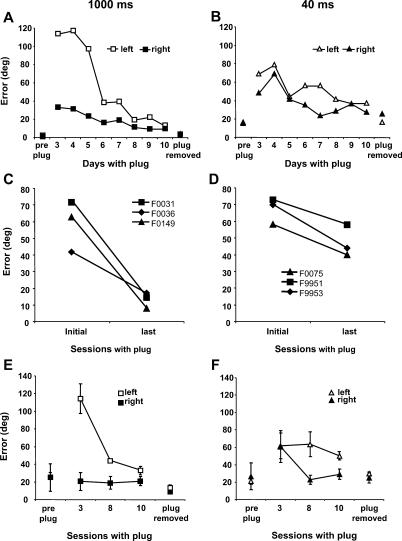
Effects of Daily Training on the Accuracy of Auditory Localization by Ferrets Fitted with Unilateral Earplugs (A) Magnitude of the unsigned errors made by one representative ferret (F0031) in response to 1,000-ms noise bursts. The errors have been averaged across speaker location in the left (plugged) and right (unplugged) hemifields. Data are shown for the session prior to insertion of the earplug, for eight daily sessions with the left ear occluded, and for the first session following plug removal. (B) Equivalent data for another ferret (F0075), whose performance was measured with 40-ms noise bursts. (C) Mean errors (across all speaker locations) at the start and end of the plugging period for three animals that were trained to localize 1,000-ms noise bursts. (D) Mean errors at the start and end of the plugging period for three different ferrets that were trained to localize 40-ms noise bursts. (E and F) Head-orienting errors (mean ± SEM) for the same animals whose approach-to-target responses are shown in (A) and (B), respectively.

In the data presented so far, auditory performance was assessed by training the animals to approach the source of the sound to receive a reward. This is the most common method used for measuring sound localization in animals [ 26, 27]. Because of the limited number of response alternatives available (in this case, 12 loudspeaker/reward-spout combinations), this is essentially a “categorization” task in which the subject presumably selects the stimulus location that most closely matches the actual perceived direction of the sound source [ 2]. We eliminated the possibility that the ferrets were learning to associate particular stimuli with specific reward spouts for the following reasons. First, the broadband noise stimuli were generated afresh on every trial and, within a given session, presented at a range of sound levels that were varied randomly. Second, as reported above, the animals could adapt to the altered spatial cues regardless of whether they were rewarded for licking any of the 12 reward spouts or just the spout associated with the speaker from which the stimulus had been presented. Moreover, in addition to the approach-to-target responses, we obtained an absolute indicator of sound localization by measuring the accuracy of the initial unconditioned head-orienting movements made by the ferrets following stimulus presentation.

Head-orienting errors also increased in magnitude in all ferrets after the left ear was occluded and then declined during the period of monaural occlusion ( *F*_1,4_ = 206.72, *p* < 0.01). We observed no effect of stimulus duration ( *F*_1,4_ = 2.897, *p* = 1.64), with all animals showing some improvement during the period in which the ear was occluded. This is illustrated in Figure 3E and 3F for two of the animals that were tested daily (the same animals whose approach-to-target responses are shown in Figure 3A and 3B, respectively). Like the approach-to-target responses, head-orienting accuracy with 1,000-ms noise bursts was disrupted primarily on the side of the plugged ear and recovered with further testing ( Figure 3E). The magnitude of the errors made in response to 40-ms stimuli increased on both sides when the ear was first occluded, but again declined with further testing despite the continued presence of the earplug ( Figure 3F).

Before the ear was plugged, the latency of the head movements in response to 1,000-ms stimuli was 175 ± 37 ms (mean ± SD). The final head bearing before the animal left the start platform to approach the sound source was reached at 627 ± 114 ms after stimulus onset. Consequently, the head was moving during these long stimuli. However, monaural occlusion had no effect on either head-orienting latency ( *F*_2,14_ = 2.67; *p* = 0.10) or the time taken for this first head turn to be completed ( *F*_2,14_ = 0.84; *p* = 0.92). The very close similarity between the accuracy of the approach-to-target and head-orienting responses with 1,000-ms noise bursts suggests that the animal's localization judgment was made during the early part of the stimulus and that once the head turn had been completed, this decision was not usually altered during the remainder of the stimulus. Moreover, because adaptation was also observed with 40-ms stimuli, which were over well before the animal started to move, we can be confident that the steady improvement in auditory localization in ferrets experiencing abnormal spatial cues is not simply based on the animals learning to track the long-duration sounds.

Daily testing consistently resulted in faster and more complete adaptation than in the animals that were tested every 6 d ( *F*_1,12_ = 14.48, *p* < 0.01; Figure 4A), even though the overall number of trials performed was the same in each group. By contrast, if after occluding one ear we waited for 6 wk before testing the animals again, we observed no improvement in their auditory localization scores, which remained at the same low level achieved when the earplug was first inserted ( Figure 4A and 4B). The consistency in the performance of these animals is also observed in normal ferrets tested over intervals of several weeks [ 28] and indicates that they were able to remember how to do the task. However, adaptation to the altered spatial cues, and therefore an improvement in performance, within this time frame required the earplugged animals to be engaged in the sound-localization task. Furthermore, the extent and rate of improvement were determined by the frequency of training in using the altered localization cues rather than by the number of trials performed or the duration of monaural occlusion.

**Figure 4:**
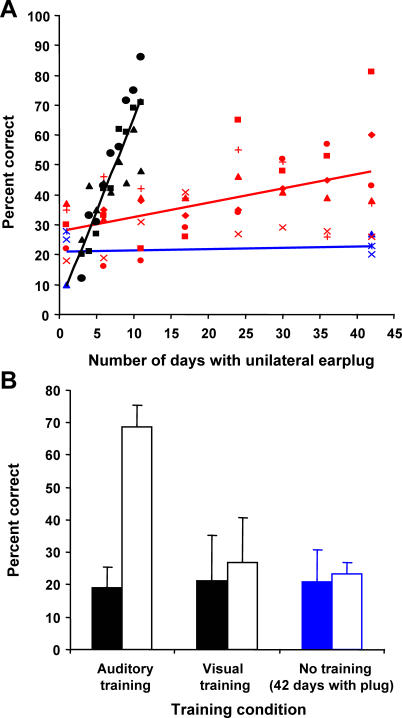
Auditory Localization Adaptation Is Accelerated by Modality-Specific Training (A) Change in performance (averaged across all speaker locations) over time in three groups of ferrets with unilateral earplugs. Each symbol represents a different animal. No change was found in trained ferrets that received an earplug for 6 wk, but were tested only at the start and end of this period ( *n* = 3, blue symbols and regression line). Two groups of animals received an equivalent amount of auditory training while the left ear was occluded. Although the earplug was in place for less time, a much faster rate of improvement was observed in the animals that received daily training ( *n* = 3, black symbols and regression line) compared to those that were trained every 6 d ( *n* = 6, red symbols and regression line). (B) Mean (± SD) auditory localization scores at the start (filled bars) and end (open bars) of the plugging period for the animals that received daily auditory training for 10 d (black symbols in [A]), another group of auditory-trained ferrets that performed a visual localization task every day while the left ear was occluded for the same period ( *n* = 3), and the three ferrets that received no training during a 6-wk period of monaural occlusion (blue symbols in [A]). The only significant improvement in sound localization was found for the earplugged animals that received daily auditory training (F _2,6_ = 15.097, *p* < 0.01).

In order to investigate the specificity of the training required to produce adaptation, we trained another group of ferrets to localize both auditory and visual stimuli, which were presented on different trials within the same session (see Materials and Methods). After collecting baseline data for each stimulus modality, we plugged the left ear and confirmed that their auditory localization ability was severely disrupted. We then provided the animals with daily training on the visual task alone for 10 d. At the end of this period, we retested their auditory localization ability and found this to be unchanged from that seen immediately after the earplug was inserted ( Figure 4B). This lack of adaptation is in stark contrast to the pronounced improvement in performance of the animals that received an equivalent amount of auditory training during this period ( Figure 4B). Learning in the sound localization task therefore appears to rely exclusively on auditory training, reinforcing our finding that adaptation is not dependent upon a visual recalibration of auditory space.

### Aftereffect following Restoration of Normal Binaural Inputs

If auditory-localization adaptation is based on a reinterpretation of the relationship between binaural cues and directions in space, restoration of a normal binaural input should produce an aftereffect toward the side of the previously plugged ear. Before the earplug was introduced, no bias was seen in the responses of the ferrets that subsequently adapted with daily training to altered binaural cues ( Figure 5A– 5C; *p* = 0.49, Wilcoxon matched-pairs signed-rank test). However, after the earplug was removed, the animals exhibited a bias toward the side of the previously plugged ear ( Figure 5D– 5F; *p* < 0.01). The aftereffect was particularly apparent for the frontal 120°; the errors made in this region of space were almost exclusively toward the left side ( *p* < 0.01), as indicated by the dots below the diagonal line in Figure 5D– 5F. An aftereffect was also observed for the animals that were tested every 6 d during a 42-d period of monaural occlusion. No bias in responses was observed in these animals prior to plugging ( *p* = 0.36), but after the earplug was removed, their responses were initially shifted toward the side of the previously plugged ear ( *p* = 0.01).

**Figure 5:**
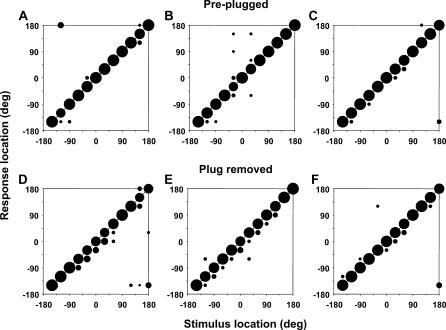
Aftereffect following Earplug Removal (A–C) Stimulus-response plots for three animals prior to insertion of the earplug. The stimuli were 1,000-ms noise bursts. The earplugs were left in place for 10 d, during which the animals underwent auditory-localization training every day (responses shown in Figures 3 and 4). (D–F) Stimulus-response plots for the same three animals for the session immediately following plug removal.

Although consistently observed following auditory adaptation, the aftereffect was transient, usually disappearing after the first session following earplug removal, and was much smaller than the bias in the opposite direction produced by the introduction of the earplug (see Figure 1). Moreover, the changes underlying auditory adaptation persisted to some degree, since replugging several weeks later had a much less disruptive effect on localization performance (paired *t*-test for comparison of errors, *p* < 0.05 at both stimulus durations), which recovered more completely than when the earplug was first introduced ( Figure 6).

**Figure 6:**
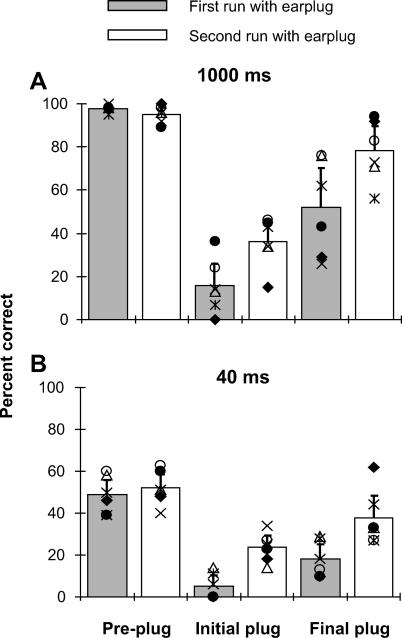
Effects of Replugging the Same Ear Mean (± SD) auditory localization scores before the left ear was occluded (“Pre-plug”) and at the start (“Initial plug”) and end (“Final plug”) of the plugging period for the six ferrets that were trained every 6 d while the earplug was in place. Superimposed on these histograms are the values obtained from individual animals, as indicated by the different symbols. (A) 1,000-ms noise bursts. (B) 40-ms noise bursts. The performance of the ferrets was the same before the ear was occluded in both runs, whereas the initial deficit caused by the earplug was lower and the recovery in performance with the earplug in place more complete in the second than in the first run.

### Basis for Auditory Localization Plasticity

The relatively modest behavioral changes observed following the removal and subsequent reinsertion of the earplug ( Figures 5 and 6) imply that a systematic retuning of neurons to the altered binaural cue values is unlikely to provide the basis for auditory localization plasticity in the adult brain. The alternative possibility is that adaptation involves learning to place greater weight on other auditory spatial cues that are less affected by monaural occlusion. The earplugs used in this study attenuated sound across a broad range of frequencies (see Materials and Methods) and therefore introduced abnormal ILDs. It is also likely that they delayed the transmission of sound through the ear, particularly at low frequencies [ 29], and changed the ITDs corresponding to different sound directions. By contrast, the spectral localization cues provided by the open ear will have corresponded to their normal sound-source directions and therefore provided information that conflicted with the altered binaural cues.

To examine the contribution of the open ear to the rapid adaptation observed with daily training following monaural occlusion, we modified the spectral cues provided by this ear by applying a unilateral mold that filled its various cavities and depressions while leaving a clear passage for sound to reach the external auditory ear canal (meatus; Figure 7A). Reshaping the external ear in this fashion altered its azimuth directional transfer function ( Figure 7B), but had no effect on the ITDs available ( Figure 7C).

**Figure 7:**
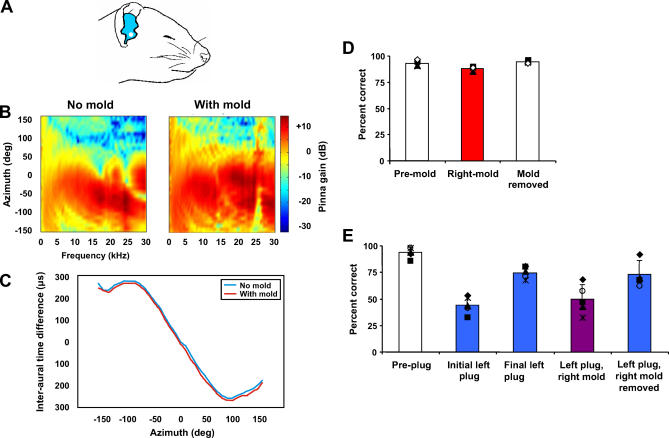
Effect of Manipulating Spectral Cues on the Accuracy of Auditory Localization (A) Drawing of a ferret's head to show the mold that was used to fill the cavities and depressions of the external ear. Note that a passage was left for sound to pass through the external auditory meatus to the eardrum. (B) Azimuth directional transfer function measured before and after the mold was fitted. (C) Variation in ITDs within the horizontal plane before and after the mold was fitted. (D) Mean (± SD) correct-score percentages for four control ferrets before the mold was fitted (“Pre-mold”), after applying it to the right ear (“Right mold”), and following its removal (“Mold removed”). (E) Mean (± SD) correct-score percentages for a group of ferrets ( *n* = 5) before the left ear was blocked with an earplug (“Pre-plug”), at the start of the 10-d plugging period (“Initial left plug”), and at the end of this period (“Final left plug”). Note that the scores improved with daily training to just below the level achieved prior to monaural occlusion. At this point (with the earplug still in place in the left ear), a mold was applied to reshape the right external ear (“Left plug, right mold”) and then removed the following day (“Left plug, right mold removed”). Superimposed on these histograms are the values obtained from individual animals, as indicated by the different symbols.

Application of a unilateral mold had a small but nonetheless significant effect on the accuracy of horizontal localization in control ferrets ( Figure 7D; *F*_2,11_ = 10.066, *p* < 0.01), consistent with this being based primarily on binaural cues. A much greater change was observed, however, when a unilateral mold was applied in order to reshape the open ear in ferrets that had previously adapted to an earplug. In these animals, localization accuracy was altered significantly by occluding the left ear and then, with the earplug still in place, by application of a mold to the right external ear ( *F*_4,23_ = 21.502, *p* < 0.01; Figure 7E). Post-hoc tests revealed significantly lower scores when the left earplug was first inserted ( *p* < 0.05), but no difference between preplug scores and those obtained following adaptation to the earplug. Applying a mold to the right ear then led to a marked impairment in performance ( *p* < 0.01), which recovered as soon as the mold was removed ( Figure 7E). Localization performance was degraded by the unilateral mold at all stimulus locations but particularly on the ipsilateral (right) side. In fact, the mean scores obtained when a mold was applied to the right ear of animals that had previously adapted to a left earplug were no different from those recorded when they first received the earplug and were about half the magnitude of those made by the control animals in the presence of a unilateral mold.

Because the acoustic measurements involved implanting a probe tube in the wall of the external auditory meatus under general anesthesia, we did not measure how the spectral cues were altered by the mold in each animal. However, in each of five ferrets that had learned to localize accurately with the left ear occluded, applying a mold to the right ear resulted in a marked increase in both the number and magnitude of the localization errors. This implies that, in contrast to normal hearing conditions, following adaptation to a unilateral earplug, auditory localization in the horizontal plane comes to rely heavily on spectral cues.

We further investigated this possibility by comparing the ability of a group of control ferrets with that of a group of animals that had adapted to a unilateral earplug to localize 1/6-octave-wide noise bursts centered on either 1 kHz or 15 kHz. Broadband noise bursts were used when these animals were learning to carry out the localization task and also when training the experimental animals to adapt to the earplug. By switching to narrowband stimuli once they had adapted, we were able to eliminate most of the spectral information but preserve binaural cues over restricted frequency ranges. The center frequencies were chosen on the basis of the ferret's head-related transfer function [ 30] to provide ITDs at a value of 1 kHz and ILDs at 15 kHz.

According to audiometric measurements, ferrets are almost equally sensitive to 1-kHz and 15-kHz tones [ 31], and both narrowband stimuli should have been above threshold at all sound levels used, even in the presence of the earplug. However, to ensure that any differences in performance between the two groups were not due to certain combinations of stimulus level and position being inaudible to the earplugged animals, we analyzed only the responses to stimuli presented at the higher levels of 70–84 dB sound pressure level **.**

Overall, both groups of ferrets localized the narrowband noise bursts less accurately than the broadband noise ( Figure 8; *F*_5,23_ = 19.926, *p* < 0.01). However, a much greater reduction in performance was observed in the animals that had learned to localize broadband signals accurately in the presence of a unilateral earplug, with their narrowband scores being significantly lower than those of the control group ( *F*_3,15_ = 14.123, *p* < 0.01). These data therefore indicate that by adapting to a unilateral earplug, ferrets become more dependent on the localization cues specific to broadband sounds.

**Figure 8:**
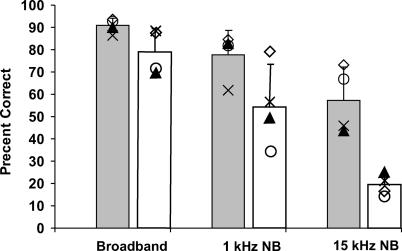
Effects of Auditory Adaptation on the Ability of Ferrets to Localize Narrowband Noise Bursts Mean (± SD) correct-score percentages for four control ferrets (gray bars) and four animals that had learned with daily training to localize broadband noise after blocking the left ear with an earplug (open bars). Data from individual animals are shown by the different symbols. The scores obtained following adaptation are shown for the broadband stimuli used for training and for 1/6-octave-wide noise bursts centered at either 1 kHz or 15 kHz. Note that while all animals achieved lower scores with the narrowband (NB) stimuli, the animals that had adapted to an earplug performed particularly poorly at the high center frequency.

Comparison of the performance at the two narrowband stimuli revealed that both control and earplugged ferrets localized sounds with a low center frequency more accurately than those with a high center frequency ( Figure 8). However, this difference was particularly pronounced in the ferrets that had adapted to a unilateral earplug, whose high-frequency scores were only slightly above the level expected by chance. These data further suggest that in learning to localize broadband sounds in the presence of the earplug, these animals were no longer using high-frequency ILDs.

## Discussion

The acoustical differences arising from variations in the size, shape, and separation of the two ears indicate that the neural mechanisms underlying auditory localization have to be calibrated by experience of the spatial cues available to individual listeners. This plasticity is most pronounced during development, when the cues are changing in value as a natural consequence of growth of the head and external ears [ 10– 14]. Our findings confirm the results of behavioral studies in humans [ 16, 17, 19– 21] in showing that the mature auditory system is also capable of adapting to markedly altered spatial cue values, and demonstrate that this plasticity can be greatly accelerated if stimulus-specific behavioral training is provided. Indeed, the extent and rate of adaptation are determined by the frequency of training, indicating that training has more benefit if concentrated into a shorter period of time. Moreover, we found that auditory localization plasticity does not depend on vision or error feedback and involves learning to place greater weight on acoustic cues that are largely unaffected by the earplug.

Our measurements of auditory localization were based on the initial head-orienting responses made by the ferrets following stimulus presentation, as well as the accuracy with which they then approached the sound source. Because we observed essentially the same pattern of adaptation with both measures of performance and with stimuli of different duration, we can rule out the possibility that the improvement in auditory localization in the presence of a unilateral earplug relies on subsequent head movements or the animals learning to track the sound.

Behavioral studies in primates and carnivores have shown that ablation or inactivation of the auditory cortex impairs the ability of these animals to approach the location of a sound source (e.g., [ 28, 32– 34]). By contrast, orienting responses, although often abnormal, are still present [ 28, 35, 36] and are generally thought to be mediated more by subcortical mechanisms. Given this dissociation, our finding that both measures of sound localization ability exhibit a comparable degree of plasticity implies that spatial processing at different levels of the auditory system can be affected by training. Other studies have shown that adult perceptual learning is associated with changes in the response properties of neurons in the auditory cortex [ 37– 42]. Experience-driven modification of subcortical circuits could potentially be mediated by descending cortifugal pathways, which Suga and Ma [ 43] have proposed might be involved in learning-induced plasticity.

### Basis for Adaptation

Raising barn owls [ 13, 44] and ferrets [ 12, 14] with one ear occluded leads to a compensatory shift in the map of auditory space in the superior colliculus, with the result that its alignment with the visual map in this multisensory midbrain nucleus is preserved. In owls, this developmental plasticity involves a shift in the tuning of the neurons for binaural localization cues [ 45, 46]. Consequently, earplug removal shifted the auditory spatial receptive fields toward the side of the previously plugged ear [ 13, 44]. Although we observed a significant aftereffect following restoration of a normal binaural input, this was much smaller and more transient than expected had the animals formed a new association between the cues distorted by the earplug and sound-source location. Consequently, it is likely that adaptation involves learning to ignore the altered cues and instead attending more selectively to cues that are unaffected by the earplug.

Direct evidence for this is provided by our finding that modifying the spectral cues provided by the open ear reduced the localization accuracy of ferrets that had adapted to occlusion of the other ear to the level observed when the earplug was first introduced. Because the same unilateral manipulation of the spectral cues had a much smaller effect on the performance of control ferrets, this result indicates that the ferrets had learned to use spectral cues for localization in a region of space, namely the horizontal plane, where they normally make relatively little contribution other than in resolving front–back ambiguities in the binaural cues [ 47, 48]. This is further supported by our finding that ferrets that had adapted to a unilateral earplug localized narrowband sounds, in which spectral cues are impoverished, particularly poorly compared to control animals.

Behavioral evidence for plasticity in the neural processing of spectral localization cues is also provided by the observation that adult humans can relearn to localize in elevation within a few weeks of altering these cues by using molds to reshape either one [ 21] or both external ears [ 19]. The pattern of adaptation observed by Van Wanrooij and Van Opstal [ 21] to a unilateral mold suggests that the auditory system can learn to use novel spectral cues independently for each ear in order to localize sound in the vertical plane. This is consistent with the possibility that ferrets adapt to a unilateral earplug by becoming more reliant upon the monaural spectral cues provided by the contralateral ear for localization in the horizontal plane.

However, reshaping the external ear with a mold will also alter interaural spectral cues, and other evidence indicates that monaural cues are by themselves insufficient to account for the localization performance achieved by the earplugged ferrets. Horizontal localization using one ear alone is usually very poor in ferrets [ 49] and humans [ 50– 53], suggesting that some input from the occluded ear is required in order for adaptation to take place. Following adaptation, the animals were able to localize low-frequency narrowband noise bursts disproportionately more accurately than high-frequency sounds, indicating that the animals are likely to use ITDs for this purpose.

It therefore appears that the rapid and pronounced behavioral adaptation revealed in this study does indeed involve a reweighting of different spatial cues. The animals learn to ignore misleading cues, specifically high-frequency ILDs, with their localization responses becoming dominated by a combination of spectral cues and low-frequency ITDs. The mechanism underlying this reweighting continues to be plastic once the earplug is removed, as shown by the initial disruption in localization performance when the same ear is occluded for a second time several weeks later. Nevertheless, reinserting the earplug resulted in significantly smaller errors, and more complete adaptation, than when it was first introduced, indicating that the physiological changes that take place during the initial phase of adaptation persist to some degree, so that subsequent disruption of binaural cues can be accommodated more effectively.

Elucidation of the physiological basis for adaptation first requires the neural locus of plasticity to be identified. As discussed above, this form of behavioral plasticity is likely to involve both the auditory cortex and subcortical processing levels. Virtual acoustic space stimuli [ 1]—which allow individual localization cues to be manipulated independently—could then be used to investigate whether neuronal changes in sensitivity to particular cues take place as an animal adapts to a unilateral earplug.

### Feedback and Auditory Plasticity

Auditory spatial learning is presumably driven by feedback about the difference between actual and perceived stimulus location. Visual cues provide a possible source of sensory feedback about the accuracy of acoustically guided behavior and could therefore guide the adaptive plasticity of the auditory localization system. Indeed, there is extensive evidence that vision can recalibrate auditory localization, both during infancy [ 14, 23, 24] and in later life [ 24, 54– 57]. Knudsen and colleagues have previously examined the role of vision in adaptation to altered binaural cues by young barn owls. Although monaural occlusion during early development can induce adaptive adjustments in the tuning of midbrain neurons to binaural cues in the absence of vision, these changes are less complete than those observed in sighted birds [ 58]. Moreover, when deprived of vision, owls are unable to recover normal localization behavior following earplug removal [ 22].

In addition to being able to localize sound just as well as control animals that could see, we found that the visually deprived ferrets adapted to an earplug just as readily. Thus, not only can accurate and even supranormal [ 59– 61] sound localization develop in the absence of vision, our results show that visual feedback is also not required for recalibrating auditory space in adulthood. It is possible that this reflects the plasticity of auditory spatial processing that results from a long-term loss of vision, and that the auditory adaptive capabilities of animals raised with an intact visual system might show more dependence on vision. Nevertheless, it is clear that auditory adaptation is possible in the absence of visual feedback.

We also found that feedback about response accuracy was not required, either in sighted or visually deprived ferrets, in order to see a progressive recovery of auditory localization in earplugged ferrets. This is consistent with studies in humans that have shown that improvement through training in sensory discrimination tasks is possible without error feedback [ 62, 63]. It therefore seems likely that the remaining, unaltered auditory cues provide sufficiently reliable information to form a basis for the relearning of accurate sound localization by the earplugged ferrets and that stimulus-specific training enhances the neural processing of these cues.

### Perceptual Learning and Auditory Localization

We found that monaural occlusion for 6 wk without training did not result in auditory localization adaptation, despite daily exposure to normal animal-house sounds, as the animals localized just as poorly at the end of this period as they did when the ear was first occluded. In contrast, juvenile ferrets raised in exactly the same environment do adapt without training, as do adult animals if the altered auditory spatial cues are experienced for a much longer period [ 12]. Thus, the conditions in which the animals were kept did provide sufficiently rich sensory stimulation to enable learning to take place. The principal finding of the present study is that, with stimulus-specific behavioral training, adaptive changes in auditory localization responses can occur in adult animals much more quickly, within a few days rather than months. A related finding has recently been made by Bergan et al. [ 57], who showed that greater plasticity is possible in the midbrain auditory space map of adult barn owls that are allowed to hunt live prey compared to birds provided with dead mice.

This dependence on training implies that rapid adaptation to altered auditory spatial cues by adult ferrets represents a form of perceptual learning. Unlike other examples of perceptual learning, however, in which practice leads to improved discrimination by individuals with normal sensory inputs [ 37– 42, 64– 70], our data show that training can be used to recover function following the introduction of abnormal inputs. Given that auditory training has been used to improve temporal processing and alleviate language impairments in children [ 71, 72], these findings suggest that it should be possible to use sound-localization training to promote rehabilitation in patients whose perception of auditory space is compromised as a consequence of hearing disorders.

## Materials and Methods

### Animals

Thirty-one adult ferrets were used in this study, and were housed either singly (males) or in pairs or larger groups (females) in standard laboratory cages, which contained nesting boxes, litter material, and various toys. The animals were allowed out of their cages several times a week, so that they could explore and play with each other and with other objects in the room in which they were kept. All procedures received local ethical committee approval and were carried out under license by the UK Home Office.

### Ear manipulations

The earplugs used in this study were always applied to the left ear. The animals were sedated with medetomidine hydrochloride (domitor, 0.15 mg/kg; Pfizer, Sandwich, United Kingdom) and the external auditory meatus occluded with a foam plug (E.A.R., Boulder, Colorado, United States). The concha of the external ear was then filled with Otoform-K _2_ silicone impression material (Dreve Otoplastik, Unna, Germany) so that it was easy to tell that the earplug was in place. The sound attenuation produced by these earplugs has been estimated, using auditory brainstem response audiometry [ 73], to be ∼40 dB across a range of 1–16 kHz. Previous behavioral measurements also showed a comparable level of attenuation at 500 Hz [ 74]. Otoscopic examination was used to confirm that the ear canals were unobstructed both prior to and following monaural occlusion.

In nine ferrets, we examined the effects of modifying the spectral cues provided by the external ear. To do this, the animals were sedated, a tube temporarily inserted into the ear canal, and Otoform-A/flex ear-impression silicone (Dreve Otoplastik) injected into the concha and other cavities of the right external ear. The tube was then removed, leaving a clear passage for sound to reach the ear canal. The acoustical consequences of the mold were determined in one ferret under general anesthesia 22 μg/kg domitor plus 5 μg/kg ketamine i.p.) by measuring the azimuth directional transfer function, as described in detail in Schnupp et al. [ 75] and in Parsons et al. [ 30]. Briefly, a polythene probe tube was inserted into the wall of the external auditory meatus and attached to a Sennheiser cartridge (KE 4–211–2) (Sennheiser, High Wycombe, United Kingdom). A metal bar was attached to the skull so that the animal could be supported from behind on a small table positioned at the center of an anechoic chamber. Acoustic signals comprised 32,768-point Golay codes, played and recorded at 100 kHz using Tucker-Davis Technologies (Alachua, Florida, United States) system-2 hardware, and delivered by a KEF T27 loudspeaker (KEF Audio, Maidstone, United Kingdom) mounted on a robotic hoop system. The signal recorded by the probe microphone was passed through an antialiasing filter (7-pole elliptic filter, F _c_ 30 kHz) and digitized. The ITDs were extracted from the microphone signals by cross-correlation of the impulse responses after low-pass filtering at 4 kHz. The amplitude spectra of the transfer function were obtained for each loudspeaker position and the contributions of the speaker and microphone removed by subtracting the amplitude spectra (in dB) measured in the absence of the animal. The plots were then smoothed with a linear Gaussian filter and the directional transfer function calculated by subtracting the mean spectral transfer function (in dB) from each of the individual functions. This procedure, which removes the location-independent components of the spectral transfer function, including the microphone and speaker transfer functions, was carried out before and after a mold was applied to the external ear.

### Behavioral measurements

Details of the positive-reinforcement paradigm have been described previously [ 28, 30]. Briefly, water-restricted ferrets were trained to initiate a trial by mounting a platform and licking a waterspout at the center of a circular testing arena. This triggered the presentation of a single burst of broadband noise (low-pass filtered at 30 kHz) from one of 12 loudspeakers equally spaced around the perimeter of the arena at 0° elevation. The output of each speaker was flattened digitally and matched for sound level. Sound levels were varied randomly within a session from 56–84 dB sound pressure level. Following stimulus presentation, the animal approached its source and received a water reward for licking the spout in front of the speaker from which the stimulus had been played. In the first earplugging experiment, the ferrets received an equal amount of water for licking any of the 12 reward spouts. Incorrect responses were followed by ≤3 correction trials in which the stimulus was presented at the same location, after which a continuous stimulus was presented (none of these trials was used to assess performance). All animals were trained to a similar degree and scored >90% correct with 1,000-ms noise bursts before any of the experiments started.

In some experiments, visual stimuli comprising single light flashes were presented from a light-emitting diode mounted above each of the speakers in the frontal hemifield (±90°). These animals were trained to localize both auditory and visual stimuli by randomly interleaving noise bursts from all 12 speaker positions with light flashes of matching duration from the seven light-emitting diodes in the frontal hemifield.

The latency and accuracy of the initial head movements made by the animals were measured by tracking the position of a reflective strip attached to the head using a vertically mounted infrared-sensitive camera and video contrast detection device (HVS Image, Harlow, United Kingdom). Our software registered the x–y coordinates of the reflective strip at the onset of the sound and sampled these values at a rate of 50 Hz for the next second. They were converted to an angle relative to the initial head position. The latency of the head movement was taken as the point when the head first moved in the same angular direction over three successive trials. The software assigned a final head bearing based on two criteria: the mean of the last three data points after the peak angular acceleration had been reached and the three points prior to a consistent change in head direction, indicating that the animal had moved off the central start platform. These two values were highly consistent. The accuracy of the orienting response was determined by the difference between the final head bearing and the direction of the sound source.

All the raw data were fed into algorithms for subsequent analysis. Criteria (e.g., for head movement latency and final head bearing) were set prior to data collection, so that there was no opportunity for the person handling the data to influence the result. Similarly, the reward spout licked by the animal was registered by the software, which converted this to a correct-score percentage and error magnitude and direction for each trial within a given session.

## Abbreviations

ILD: interaural level difference
ITD: interaural time difference
